# The Role of Microbiome in Cardiovascular Health: Insights for Primary Care Interventions

**DOI:** 10.7759/cureus.70311

**Published:** 2024-09-27

**Authors:** Joanne Arvelaez Pascucci, Patricia E Ghattas, Ricardo Olivas Lerma, Sol Villa Nogueyra, María Belén Nogales Bernal, Valentina Milani, Lautaro M Floridia Rietmann, Samantha M Alvarez, Jorge Salaz Diaz

**Affiliations:** 1 Infectious Disease, Universidad Central de Venezuela, Caracas, VEN; 2 Internal Medicine, Universidad Católica de Honduras, Tegucigalpa, HND; 3 Internal Medicine, Universidad Autónoma de Chihuahua, Chihuahua, MEX; 4 General Practice, Universidad de Buenos Aires, Buenos Aires, ARG; 5 Internal Medicine, Clínica Dávila, Santiago, CHL; 6 Internal Medicine, Sapienza University of Rome, Rome, ITA; 7 Internal Medicine, Facultad de Ciencias Médicas, Universidad Nacional del Litoral, Santa Fe, ARG; 8 Family Practice, St George’s University, St. George's, GRD; 9 Internal Medicine, Universidad Nacional Autónoma de México, Mexico, MEX

**Keywords:** cardiovascular disease, gut microbiome, immune modulation, prebiotics, probiotics

## Abstract

Cardiovascular diseases (CVDs) remain the leading cause of morbidity and mortality worldwide, highlighting the urgent need for effective prevention strategies. Emerging research suggests that the gut microbiome is critical in cardiovascular health, influencing pathophysiological processes associated with CVDs. This narrative review explores the intricate relationship between the gut microbiome and cardiovascular health, mainly focusing on how microbial composition affects inflammation, lipid metabolism, and endothelial function. Additionally, we discuss the implications of gut microbiome modulation through dietary interventions, prebiotics, and probiotics as potential therapeutic strategies for primary care practitioners. By emphasizing the importance of the microbiome in cardiovascular risk management, this review aims to inform primary care interventions that leverage microbiome research to improve patient outcomes and prevent CVDs. Ultimately, understanding and integrating gut health into cardiovascular care may provide a novel approach to enhancing cardiovascular resilience and reducing disease burden.

## Introduction and background

Cardiovascular diseases (CVDs) are the worldwide leading cause of mortality and disability. They are a group of disorders of the heart and vessels and include coronary artery disease, cerebrovascular disease, and peripheral vascular disease. CVDs are responsible for 32% of global deaths, which represent approximately 17.9 million deaths every year. Of these deaths, 85% are due to myocardial infarction (MI) and stroke, and one-third occur in people under 70 years of age ​[1]. The human gut microbiota comprises diverse bacterial communities, among which 90% consist of bacteria from the phyla Firmicutes and Bacteroidetes. Firmicutes include bacteria from the genera *Faecalibacterium*, *Enterococcus*, *Streptococcus*, and *Ruminococcus*; Bacteroidetes include *Bacteroides* and *Prevotella ​*[1]. The primary roles of the gut microbiome are digestion, epithelial barrier generation, preservation against pathogens, and regulation of the immune system and inflammation [2,3]. As a result, an increased Firmicutes and Bacteroidetes ratio (F/B ratio) is related to developing diseases like obesity, diabetes, and CVD [1].

Recently, a prognosis marker known as trimethylamine N-oxide (TMAO) has been associated with the detection of CVD [1]. TMAO is an organic compound produced by gut microbiota, and its concentration in blood increases after ingesting foods rich in L-carnitine and phosphatidylcholine, like red meat, eggs, and fish. It is associated with atherosclerosis by affecting lipids transportation, foam cell development in the artery wall, and platelet hyperactivity ​[4,5]. Furthermore, TMAO is associated with endothelial dysfunction caused by cellular inflammation and oxidative stress ​[6]. 

Since the gut microbiome is one of the modifiable risk factors for CVDs, appropriate primary care interventions that manage and regulate it may help prevent future diseases. For this reason, this narrative review aims to investigate the relationship between the gut microbiome and cardiovascular health, emphasizing it in the primary care setting. 

## Review

Inflammation and immune modulation 

**Figure 1:**
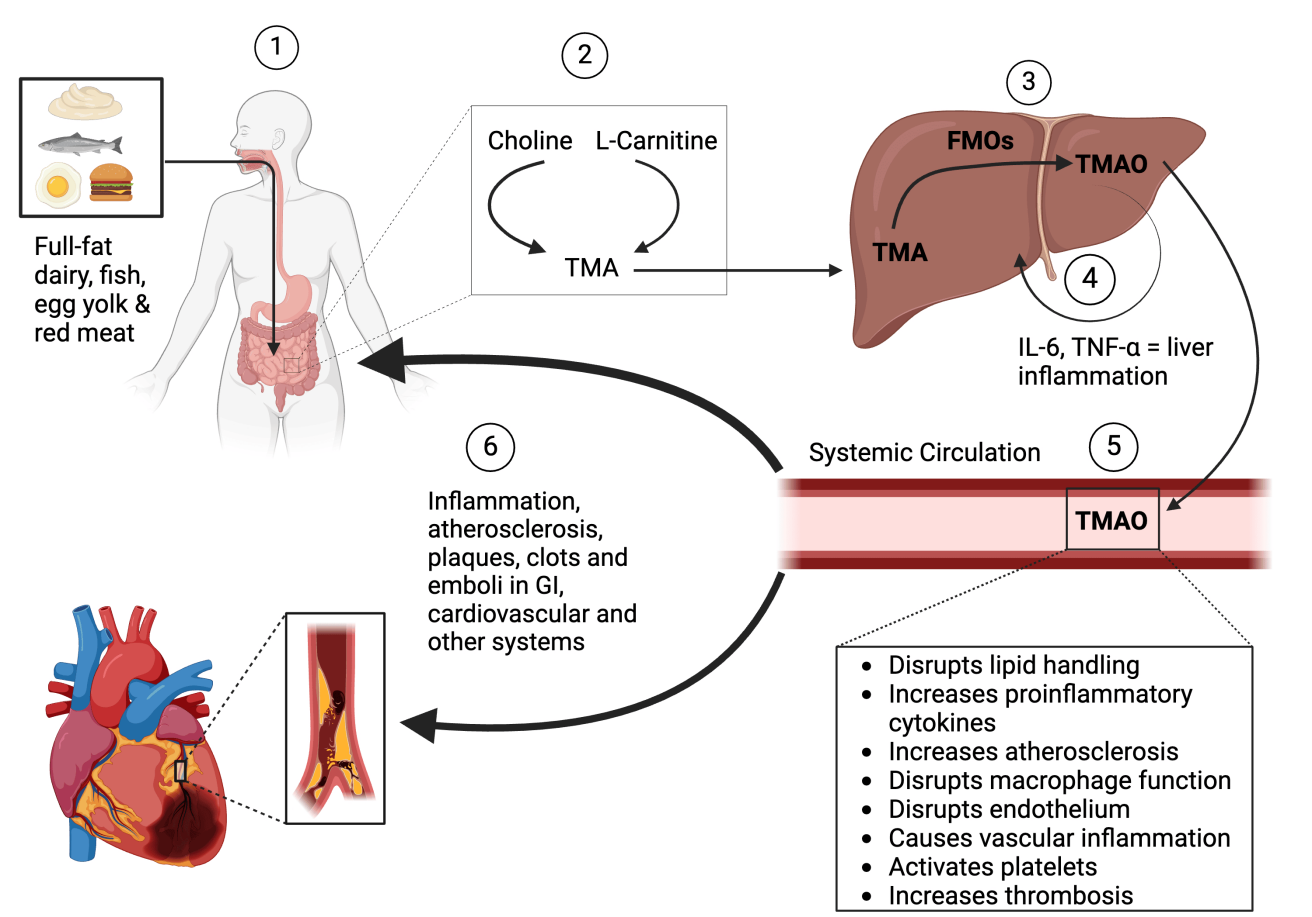
Formation, distribution, and deleterious effects of TMAO (1) The gut metabolizes certain foods, such as red meat, fish, full-fat dairy, and egg yolks [7]. (2) The choline and L-carnitine elements are used as building blocks to synthesize TMA [14]. (3) The formed TMA flows to the liver via the portal circulation, oxidizing it into TMAO by FMOs [7]. (4) TMAO directly stimulates liver inflammation through pro-inflammatory cytokines such as IL-6 and TNF-α [13]. (5) The oxidized TMAO is also released to the systemic circulation, generating inflammation and oxidative damage to the blood vessels, disrupting lipid handling and endothelial linings, and encouraging plaque, thrombus, and embolus formation [13]. (6)This leads to atherosclerotic and inflammatory damage to cardiovascular, gastrointestinal, and other body systems [13]. TMA: triethylamine; FMO: flavin-containing monooxygenase; TMAO: triethylamine-N-oxidase; IL-6: interleukin-6; TNF-α: Tumor-necrosis factor alpha. Figure created by authors with BioRender.

The gut microbiota acts like an endocrine organ by secreting metabolites that act as hormone-like signals that impact the body's normal physiology ​[7-9]. Gut microbial-derived hormones include short-chain fatty acids (SCFAs) and 4-ethyl phenyl sulfate ​ trimethylamine (TMA)/TMAO [10-13]. TMA is converted into TMAO, a proatherogenic factor, in the liver by flavin-containing monooxygenases (FMOs)​ [3,7,12], which​ promotes cholesterol accumulation within macrophages by inducing scavenger receptors such as CD36 and SRA1; both receptors are involved in the uptake of modified lipoproteins contributing to foam cell formation and the development of atherosclerosis, as explained in Figure 1​ [1,7]. 

TMAO has been associated with poor outcomes of primary sclerosing cholangitis (PCS), affects the metabolism of hepatic and intestinal cholesterol and bile acid, and produces changes in macrophage phenotype in the artery wall [1,7,14,15]. TMAO has also been associated with liver inflammation, causing elevated liver enzymes that can be confused with other diseases like non-alcoholic fatty liver disease (NAFLD) and other liver conditions. Chronic inflammation could lead to secondary complications, and as previously mentioned, inflammation is an essential process for CVD, so therapies and diseases that affect this process are crucial to decreasing CVD ​[16-19]. In more recent years, therapies that target the microbiome have been shown to reduce inflammation, showing a promising avenue for research ​[20,21].​ 

Role of inflammatory mediators in atherosclerosis 

Atherosclerosis is a chronic inflammation disease affecting large and mid-sized arteries [22,23]. This chronic condition in the vessels results in stenosis that restricts blood flow and causes tissue hypoxia, leading to cerebrovascular complications such as MI and stroke ​[24]. The disease develops when low-density lipoprotein (LDL) enters the artery wall and becomes oxidized ​[25]. Oxidized LDL (OxLDL) is recognized by immune system receptors, including scavenger receptors on macrophages, leading to foam cell formation and inflammatory cytokines secretion. The production of these substances leads to the recruitment of inflammatory cells such as monocytes, T cells, natural killer cells, natural killer T cells, and dendritic cells into the intima ​[25]. Therefore, atherosclerotic lesions of a lipid core region consist mainly of cholesterol, triglycerides, and other lipids, and the fibrous cap contains various immune cells, each contributing differently to the pathophysiology of atherosclerosis [23,24,26]. 

While natural killer (NK) cells do not play a role in the progression of atherosclerosis, other immune cells such as macrophages and T-cells are actively involved in the disease process [27,28]. Macrophage foam cells dominate in the core; TMAO promotes lipid accumulation by reducing the ATP-binding cassette A1 (abca1) pathway and oxidative stress by upregulating the Nrf2 pathway in these cells [29]. Foam cells produce a variety of cytokines and release proteolytic enzymes, resulting in decreased extracellular matrix production and enhanced apoptosis within the necrotic core. Dying macrophages will then release lipid contents and tissue factors, contributing to atherosclerosis plaque formation [30]. T-cells are found in the fibrous cap, and B cells in the perivascular adipose tissue (PVAT) [24,25,31,32]. CD8+ T cells promote atherosclerosis through two mechanisms, first by leading to vascular inflammation by the secretion of proinflammatory cytokines such as interferon‐gamma (IFN‐γ) and tumor necrosis factor-alpha (TNF-α) and second by increasing apoptotic cell numbers in lesions [23,31]. In addition, B cells contribute by stimulating TH1 adaptive immunity [23] and producing immunoglobulin G (IgG), which deposits in atherosclerotic lesions and acts against LDL and oxLDL [24,33]. 

Microbial metabolites and cardiovascular risk

**Figure 2:**
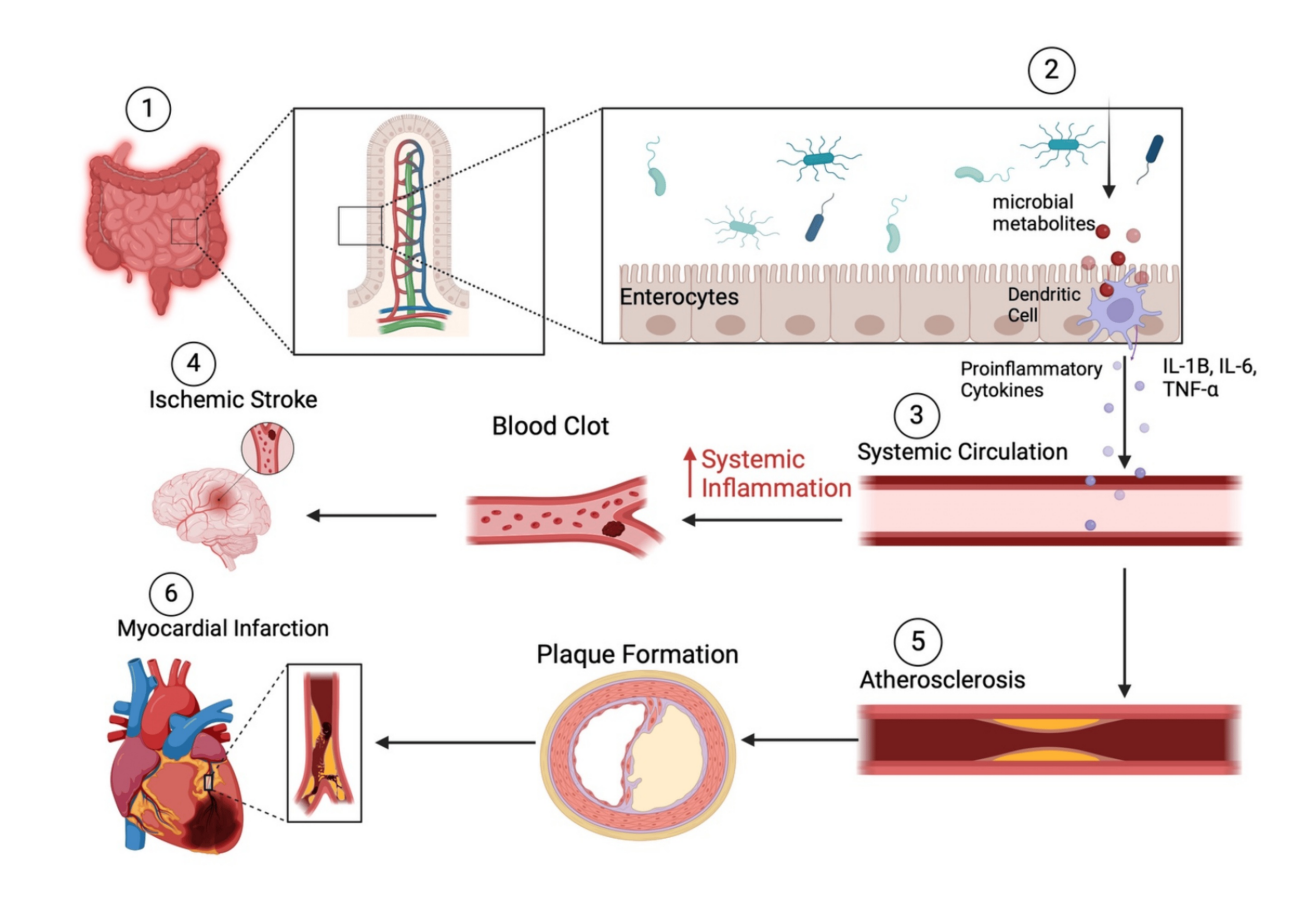
Gut biome dysbiosis and cardiovascular disease (1) Gut dysbiosis ​[7]​ consists of pathogenic, non-commensal microbes releasing metabolites into the gut lumen ​[36], (2) which then activate dendritic cells (DCs) among the enterocytes. (3) The activated DCs then release proinflammatory cytokines such as IL-1B, IL-6, and TNF-α into systemic circulation ​[23,31]. (4) This results in systemic inflammation that can lead to blood clots, leading to ischemic stroke. (5) It can also, supported by TMAO, cause and exacerbate atherosclerosis and plaque formation ​[25]. (6) Plaques can then rupture, leading to embolism and infarction within the myocardium and other oxygen-sensitive tissues ​[24]. IL-1B: interleukin-1B; IL-6: interleukin-6; TNF-α: tumor necrosis factor-alpha; TMAO: trimethylamine-N-oxide Figure created by authors with BioRender.

It has been shown that TMAO negatively impacts cholesterol metabolism by hindering reverse cholesterol transport and increasing cholesterol buildup within macrophages, leading to enhanced foam cell formation and dyslipidemia, which contribute to the development of atherosclerotic plaques ​[29]. Moreover, TMAO has been shown to impair endothelial function and provoke vascular inflammation, resulting in vascular dysfunction ​[34]. It also encourages platelet aggregation, heightening the risk of thrombosis and increasing the likelihood of atherosclerotic plaque rupture. TMAO can also extend the effects of angiotensin II-induced vasoconstriction, promoting angiotensin II-induced hypertension and causing the glomerular filtration rate (GFR) to decrease through the protein kinase R-like ER kinase (PERK)/reactive oxygen species (ROS)/calcium/calmodulin-dependent protein kinase II (CaMKII)/PLC3 pathway. CVD patients with TMAO concentrations at 5 μmol/L experienced a 7.34% increase and at 10 μmol/L, a 15.22% rise in circulating TMAO levels ​[35]. Recent robust evidence suggests that individuals with CAD tend to have higher circulating TMAO levels compared to healthy individuals, indicating that TMAO could be a potential biomarker for cardiovascular risk and a target for therapeutic intervention in individuals with CAD​ as explained in Figure 2 [5,7,23-25,31,36]. 

SCFAs are primarily produced through the fermentation by the gut microbiota in the cecum and colon of indigestible saccharides like dietary fibers, non-starch polysaccharides, or resistant starches with acetate, propionate, and butyrate compromising about 95% of SCFAs in an approximate molar ratio of 3:1:1 within the intestinal lumen [37]. The remaining less than 5% is composed of formate, valerate, and caproate. Recent studies show that butyrate inhibited intestinal cholesterol absorption and promoted cholesterol excretion in intestinal cells by regulating the expression of mRNA-associated transporters [36]. This significantly lessened atherosclerosis induced by the apolipoprotein E (apoE) -/- mice diet. The apoE -/- mice, or apoE knockout mice, are genetically modified mice that lack the gene responsible for producing apoE [38].

Additionally, butyrate decreases nitrotyrosine and induces inducible nitric oxide synthase (iNOS) formation in lesion site of ApoE -/- mice and also reduces the levels of ROS in vascular smooth muscle cells by upregulating the level of glutathione-S-transferase. SCFAs also influence lipopolysaccharide (LPS) or TNF-a-induced endothelial activation by inhibiting the production of IL-6 and IL-8 and reducing the expression of VCAM-1 and subsequent cell adhesion ​[38]. Butyrate has been proven to decrease the release of MCP1/CCL2 and, hence, reduce the migration of monocytes to the lesion area. Accumulation of oxLDL in macrophages is a sign of early atherosclerosis as it binds to scavenger receptors such as CD36 and oxLDL. Butyrate decreases CD36 expression in peritoneal macrophages from apoE -/- mice stimulated by oxLDL. These mechanisms suggest that SCFAs exhibit anti-atherosclerotic properties by decreasing monocyte adhesion, preventing cholesterol aggregation, and reducing macrophage inflammation and foam cell formation ​[39]. 

Approximately 95% of bile acids are reabsorbed in the ileum and cycled back to the liver, while the remainder reaches the colon, where they are metabolized by gut microbiota. This microbial metabolism converts primary bile acids into unconjugated forms via microbial cholesterol 7α-dehydroxylation. The mechanisms mediated by bile acid receptors have been implicated in regulating bile acid biosynthesis, lipid metabolism, atherosclerosis, and the metabolic abnormalities associated with CVD ​[7].

Intestinal barrier function and endotoxemia

As previously stated, the gut is a biological niche home to diverse microbes ​[40]. The composition of this niche is shaped by various environmental factors such as predator bacterial communities, which prevent the overgrowth of predominant bacterial species, pH, and anaerobic conditions of the gut, as well as our diet ​[41]. Microbial diversity differences correlate with mucosa inflammation and gut permeability ​[42]. Of the gut microbiota, 90% consists of Firmicutes and Bacteroides, as mentioned previously ​[1]. Abnormal changes in this population, like an increased F/B ratio [5], also known as gut dysbiosis, are associated with CVDs ​[36]. Gut dysbiosis increases gut permeability, leading to high levels of LPS ​[41-45]. LPS is a bacteria's membrane component that promotes inflammation by activating the immune system ​[46]. Cell activation, proliferation, and secretion of proinflammatory cytokines like IFN-γ, TNF-α, IL-2, IL-3, and lymphotoxin (LT) can promote atherosclerosis by maintaining chronic inflammation and inducing foam cell formation ​[20,42,43]. 

Recent studies emphasize the substantial impact of the intestinal microbiome on the risk of chronic diseases, particularly CVD. They highlight that diet, which can rapidly alter the microbial composition, is crucial in shaping the microbiome and offers potential therapeutic benefits ​[46, 47]. 

Several studies have recognized an essential link between SCFAs, TMO, and CVD, as shown in Table 1 [48-57].

**Table 1:** Key studies on the gut microbiome and CVD TMAO: trimethylamine-N-oxide; MACE: major adverse cardiovascular events; CVD: cardiovascular disease; RCTs: randomized controlled trials; HF: heart failure; F/B: Firmicutes/Bacteroidetes; MACE: major adverse cardiovascular events; MACCE: major adverse cardio and cerebrovascular events; ACVD: atherosclerotic cardiovascular disease; SCFA: short chain fatty acids; CAD: coronary artery disease; RR: relative risk; BCAAs: branched-chain amino acids; hs-CRP: high-sensitivity C-reactive protein; IL: interleukin

Study	Population	Key Findings	Relevance to Primary Care
Heianza et al. (2017) [48]	A total of 19 prospective studies with quantitative estimates of the associations of TMAO with the development of MACE or death.	Higher circulating levels of TMAO and its precursors are associated with an increased risk of MACE.	Basic dietary modifications and gut microbiome-focused therapy that reduces TMAO levels are suggested to prevent and treat CVD.
Huang et al. (2024) [49]	A total of 15 studies were included with patients with and without HF, The studies included RCTs, non-RCTs, open trials, cross-sectional trials, or prospective trials in which gut microbiota analysis was performed and diversity or abundance measures were reported, They were published as full-text articles in peer-reviewed scientific journals.	Higher abundance of Proteobacteria and Actinobacteria in HF group, while F/B ratio and Bacteroidetes are lower. There is an increase in the relative abundance of *Streptococcus, Bacteroides, Alistipes, Bifidobacterium, Escherichia-Shigella*, *Enterococcus* and *Klebsiella *in the HF group, whereas *Ruminococcus, Faecalibacterium, Dorea*, and* Megamona* show a decrease in relative abundance.	New strategies for heart failure prevention and treatment can be developed using microbiota analysis.
Schiattarella et al. (2017) [50]	A total of 17 clinical studies were included in the analytic synthesis, enrolling 26,167 subjects. Associations between TMAO plasma levels, all-cause mortality (primary outcome) and MACCE (secondary outcome) were systematically addressed.	Data presented in this study showed a significant positive dose-dependent association between plasma TMAO levels, cardiovascular events, and mortality.	The modulation of TMAO levels or its precursors might represent a novel potential therapeutic approach to change CVD prognosis or the use of TMAO as a biomarker for cardiovascular risk.
Van den Munckhof et al. (2018) [51]	A systematic review of 14 studies including healthy subjects or with obesity/overweight	Obesity and overweight are known as a low-grade pro-inflammatory chronic state. Lower gut microbial diversity was associated with higher white blood cell counts and hsCRP levels. The abundance of Bifidobacterium, Faecalibacterium, Ruminococcus and Prevotella were inversely related to different markers of low-grade inflammation such as hsCRP and IL-6.	The gut microbiota could serve as a diagnostic marker by which a more pro-inflammatory state could be detected in an early stage and could predict the risk of developing certain ACVD states. Potentially, the use of prebiotics, probiotic strains, or faecal microbiota transplantation via capsules could become a promising therapeutic option to prevent low-grade inflammation and ACVD in the future.
Simadibrata (2023) [52]	Peer-reviewed human studies comparing the gut microbiota profile in adult patients with HF and healthy controls	Gut microbiota diversity, richness, and composition in HF patients differ significantly from the healthy population.	Depletion in SCFA-producing gut bacteria in HF patients may contribute to changes in immune modulation and neuroenteroendocrine hormone imbalance; therefore, affecting the progression or worsening of HF.
Hamjane et al. (2024) [53]	Systematic review of 150 articles	Gut microbiota dysbiosis plays a crucial role in the development of various obesity-related metabolic abnormalities, among them type 2 diabetes and CVD	Intestinal microbiota appears to be a promising target for the nutritional or therapeutic management of these diseases.
Choroszy et al. (2022) [54]	A total of 21 articles met inclusion criteria for systematic review and seven for meta-analysis	The alterations in the gut microbiota composition are associated with qualitative and quantitative changes in bacterial metabolites, many of which have pro-atherogenic effects on endothelial cells, increasing the risk of developing and progressing CAD.	The gut microbiota composition can be modulated by an appropriate diet. Targeting personalized medicine with dietary selection or supplementation with beneficial bacterial species could help reduce cardiovascular morbidity and mortality.
Ge et al. (2020) [55]	A total of eight studies with 11,750 individuals and 6176 hypertensive cases	Suggests a significant positive dose-dependent association between the circulating TMAO concentration and hypertension prevalence regardless of different stratifications. The RR for hypertension prevalence increased by 9% per 5-μmol/L increment, and by 20% per 10-μmol/L increment in circulating TMAO concentration.	Modulation of TMAO concentrations could lead to a better hypertension prognosis and with this lower CVD risk.
Agnoletti et al. (2022) [56]	A total of 24 articles from three databases, of which 12 were based on animal studies and 12 on humans, and the relationship between vascular ageing and gut microbiota was studied.	Identiﬁed two association patterns, consistently present in most animal and human studies: (I) a direct correlation between arterial stiffness and abundances of bacteria associated with altered gut permeability and inﬂammation (mainly from the Clostridium genus), as well as with biological markers of inﬂammation; (II) an inverse relationship between arterial stiffness, microbiota diversity, and abundances of bacteria associated with most ﬁt microbiota composition (butyrate producers, Akkermansia, Biﬁdobacterium, Ruminococcaceae, Faecalibacterium, Lactobacillus).	Vascular ageing can precede hypertension and organ damage and is associated with cardiovascular risk, identifying primary care prevention therapeutics that modify gut microbiota can reduce morbimortality of CVD.
Sanchez-Gimenez et al. (2023) [57]	21 studies involving 58,691 participants, 14 articles included participants free of CVD at the baseline and 7 articles participants had at least one CVD condition.	TMAO was positively associated with adverse cardiovascular events and CVD/all-cause mortality in some, but not all of the included studies. Bile acids were associated with atrial ﬁbrillation and CVD/all- cause mortality, but not with CVD. Positive associations were found between BCAAs and CVD, and between indole derivatives and MACE while a negative association was reported between tryptophan and all-cause mortality. No studies examining the relationship between SCFAs and CVD risk were identiﬁed.	Future strategies for reducing and treating CVD.

Vitamins are crucial for heart health; they can impact the risk and progression of CVD, with specific benefits like vitamin D for calcium absorption, vitamin B1 for energy metabolism, vitamin B6 and Vitamin A for erythrocyte production, vitamin E for antioxidant protection, and vitamin K for blood clotting and vessel integrity While the role of multivitamins in preventing chronic diseases remains debated, some vitamins may provide unique advantages for cardiovascular health. The relationship between vitamins, minerals, and gut microbiota is complex, each influencing the absorption and metabolism of the other, highlighting the need for further research [58,59]. For instance, consumption of plant-based proteins has been associated with increased beneficial gut bacteria like *Bifidobacterium* and *Lactobacillus* while simultaneously reducing harmful bacteria such as *Bacteroides fragilis* and *Clostridium perfringens* ​[60]. Conversely, diets rich in animal-based proteins are linked to elevated levels of bile-tolerant anaerobes including *Bacteroides*, *Alistipes*, and *Bilophila* ​[61]. Furthermore, specific microbial genera that proliferate with red meat consumption, such as *Ruminococcus*, are connected to increased levels of TMAO. This compound promotes atherosclerosis and heightens the risk of CVD ​[62]. 

Regarding carbohydrates, the intake of non-digestible carbohydrates found in whole grains has been linked to a decrease in proinflammatory cytokine IL-6, insulin resistance, and peak postprandial glucose levels. This dietary pattern has also been associated with reductions in total body weight, serum triglycerides, total cholesterol, LDL cholesterol, and hemoglobin A1c concentrations ​[62]. When considering fats, diets high in saturated and trans fats are believed to raise the risk of CVD by increasing blood levels of total and LDL cholesterol ​[63]. Evidence suggests that lowering dietary fat intake while increasing carbohydrate consumption can enhance the fecal abundance of Bacteroides and Bifidobacterium spp., bacterial groups independently associated with better energy regulation and reduced metabolic syndrome risk factors, such as fasting glucose and total cholesterol ​[64]. Evidence suggests that microbiota gene content, whether low (LGC) or high (HGC), significantly impacts clinical markers like lipid levels and inflammation, with dietary interventions leading to more significant improvements in the HGC group, indicating that a richer microbiome may better predict positive responses to dietary changes ​[65]. Although nutritional interventions can increase gene richness in the LGC cohort, they remain significantly lower than in the HGC group, highlighting the crucial role of microbiome richness in the effectiveness of these interventions and its close association with metabolic disease phenotypes ​[66]. 

Current research highlights that diet is a critical factor in shaping the composition and structure of the gut microbiome ​[67]. Strategies to manipulate the gut microbiota, such as using dietary supplements like probiotics and prebiotics or consuming fermented foods like kimchi, kombucha, yogurt, and sauerkraut, have gained significant attention ​[68]. These approaches have been shown to reduce inflammatory markers in healthy individuals by increasing microbial diversity and enriching beneficial short-chain fatty acid-producing species, potentially lowering the risk of inflammatory conditions like diabetes and CVD ​[69]. 

**Figure 3:**
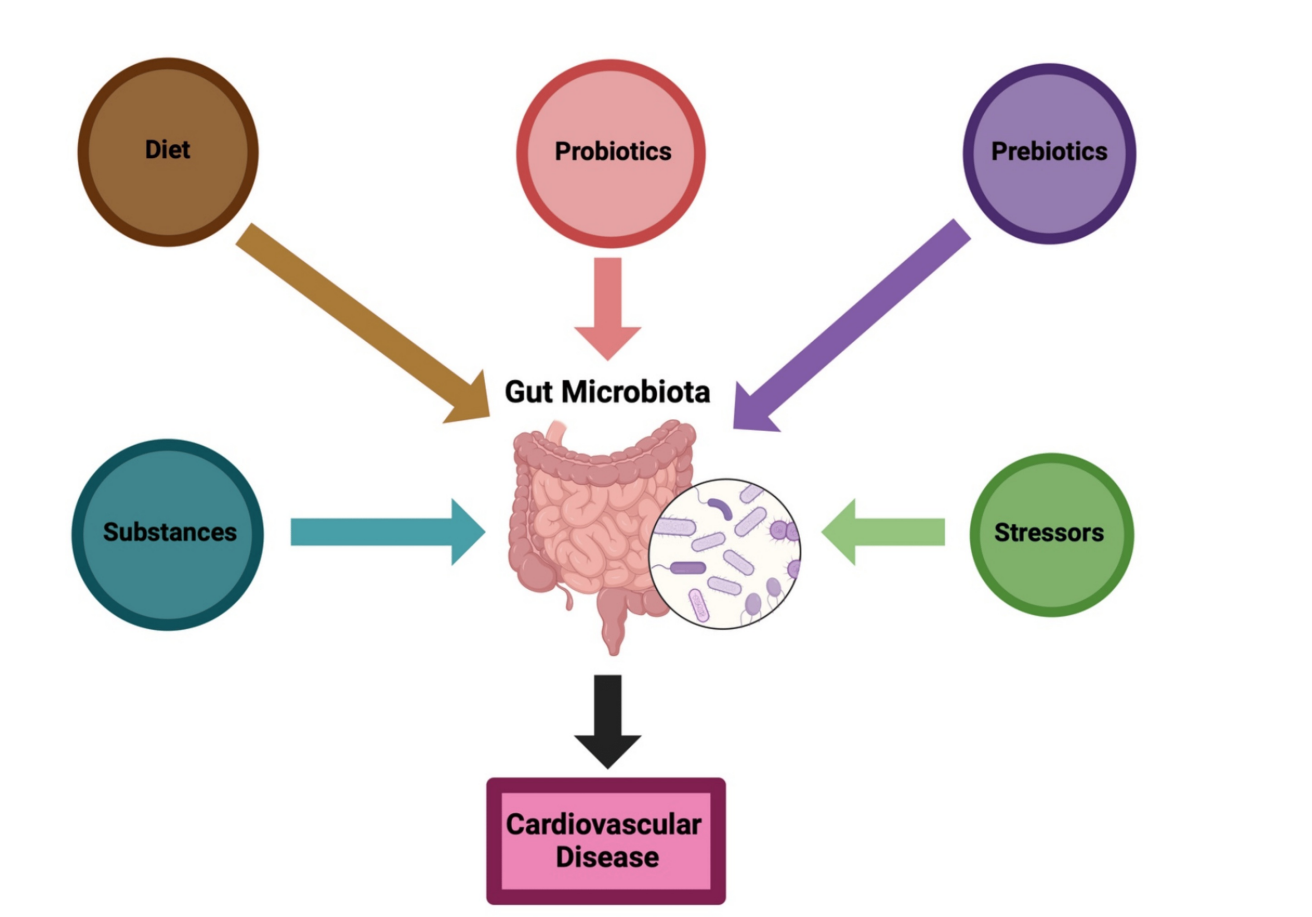
Summary of primary care interventions targeting the gut microbiome The gut microbiome is affected by diet, as would be expected based on its function [49]. It is also sensitive to consumed substances and stressors [46,60,70], and each of these can be targeted for primary care interventions to reduce gut dysbiosis and therefore cardiovascular disease [71]. For example, consuming a diet high in whole grains [43,46] and low in fats [67] can support gut eubiosis, decreasing inflammation and cardiovascular disease. Decreasing or eliminating substances and stressors that negatively affect microbiota, such as substances like tobacco and alcohol [66-72], as well as psychiatric disorders [73], can also benefit gut and cardiovascular health. Certain beneficial foods and substances can be added or increased to supplement probiotics and/or prebiotics, balancing gut health and inflammation [41,74]. Figure created by authors with BioRender.

Beyond diet, several lifestyle factors significantly influence microbiome health and are established risk factors for CVD. Tobacco and alcohol use, for instance, negatively affect the microbiota, contributing to a pro-inflammatory environment that heightens CVD risk ​[60,61]. Additionally, stress and psychiatric disorders have been linked to proinflammatory changes in the microbiota, further exacerbating cardiovascular risk [​70]. These insights highlight the crucial role of primary care in addressing modifiable risk factors such as diet, tobacco and alcohol use, and stress management. By incorporating targeted lifestyle interventions into routine care, primary care providers can effectively mitigate CVD risk and improve overall cardiovascular health, making these strategies both practical and essential, as explained in Figure 3 ​[41,49,67-70].​ 

Prebiotic and probiotic supplementation

Evidence strongly suggests that daily consumption of probiotics, with doses ranging from 10^9^ to 10^12^ colony-forming units (CFU) for three to nine weeks, can lead to improvements in blood pressure (BP). The most significant benefits are observed in individuals with elevated BP, especially when the daily probiotic dose is at least 10^11^ CFU. The intervention lasts a minimum of eight weeks ​[75,76]. Beyond BP regulation, probiotics positively affect lipid metabolism by lowering total cholesterol, LDL cholesterol, and triglycerides while boosting high-density lipoprotein (HDL) cholesterol levels. 

Both probiotics and prebiotics are essential components of cardiovascular health management. Probiotics contribute to weight management by influencing energy metabolism, fat storage, and appetite regulation, which are critical for reducing cardiovascular risk ​[77]. Prebiotics support cardiovascular health by improving lipid profiles through increased SCFA production, which inhibits cholesterol synthesis and promotes bile acid excretion ​[78]. Additionally, prebiotics enhance insulin sensitivity, particularly in those with type 2 diabetes, and exhibit anti-inflammatory effects by bolstering gut barrier function and reducing endotoxin leakage ​[79,80]. The combination of probiotics and prebiotics, known as synbiotics, provides synergistic benefits, further modulating the gut microbiota and offering a comprehensive strategy for cardiovascular health management ​[80]. 

Incorporating probiotic-rich foods, such as yogurt and kefir, into dietary plans is a practical approach to enhancing cardiovascular health in primary care settings. Fermented foods like dairy products and kimchi, coupled with high-fiber prebiotic foods such as garlic and whole grains, can improve gut microbiota function, lipid metabolism, and glycemic control, thereby reducing cardiovascular risks ​[75-79]. Primary care providers can implement these strategies through patient education, personalized dietary interventions, and ongoing monitoring to achieve optimal outcomes. The accessibility of these foods, which can be easily included in daily diets, highlights the practicality of this approach [81].

Despite these benefits, further research is needed to develop public health policies that support the integration of probiotics and prebiotics into primary care. Expanding the evidence base and establishing comprehensive guidelines could significantly enhance public health outcomes [82].

Recent research underscores the significant role of nutrients in shaping the gut microbiota. Specific dietary components have been shown to selectively promote the growth of particular microbial communities, thereby enhancing their capacity to extract energy from ingested food ​[83]. Metanomic and metabolomic analyses illuminate this dynamic adaptability of the gut microbiota to dietary shifts, which reveal a nuanced balance between protein fermentation in animal-based diets and carbohydrate fermentation in plant-based diets ​[84]. 

The practicality of these dietary interventions makes them well-suited for primary care settings. Synbiotic and prebiotic-rich foods can be integrated into patients' diets with minimal resources, making this approach accessible and sustainable. Regular monitoring and evidence-based practices support using synbiotics to potentially enhance cardiovascular outcomes by modulating the gut microbiome. 

Future directions

CVD management is advancing through personalized medicine, emphasizing microbiome-based interventions. Therefore, adopting Mediterranean, vegan, or personalized dietary programs (PDPs) to manage ischemic heart disease, diabetes, and hypercholesterolemia, focusing on enhancing beneficial gut bacteria, such as *Ruminococcaceae* and *Bifidobacterium*, is crucial. These microbes positively influence lipid profiles, inflammatory markers, glucose levels, and body weight, highlighting the need for interventions tailored to gut composition and closely monitoring these parameters ​[85-87]. Merging insights suggest that specific probiotics, including *Bifidobacterium, Lactobacillus*, and *Saccharomyces boulardii*, may offer tailored interventions for managing CVD like post-MI, untreated hypertension, and chronic heart failure by reducing inflammation, regulating blood pressure, and improving heart health through careful daily monitoring and dosage adjustments ​[44,88-90]. Additionally, fecal microbiota transplant (FMT) offers a promising option for restoring gut health in metabolic disorders, especially when conventional therapies have shown recurrences​ [91].​ 

Integrating genetic profiling with microbiome sequencing can lead to more precise treatments based on genomic and microbial insights. Sing 16S RNA gene analysis can identify microbiome imbalances, allowing tailored diets and probiotics to target harmful bacteria and promote beneficial ones that impact inflammatory and metabolic markers ​[92,93]. Meanwhile, analyzing fad genes related to lipid metabolism and DNAemia levels supports nutritional interventions such as berberine, probiotics, and polyphenol-rich diets, which can complement statins and improve dyslipidemia management ​[85,86]. These innovations could tailor nutritional and microbial interventions alongside complementary pharmacological treatments, optimize management, reduce dosages, and enable proactive prevention for high-risk groups [94]. 

Despite advances, significant gaps remain that could be addressed to pave the way for more personalized and effective cardiovascular treatments. While many studies focus on complex, hospital-based interventions, simpler, non-invasive methods like diet and probiotics, mainly when tested on larger sample sizes, could significantly enhance treatment personalization and effectiveness. Large-scale trials are needed to validate these methods, assess their long-term impacts, and ensure their efficacy across diverse populations ​[95]. Also, healthcare professionals must stay current with emerging therapeutics and evaluate treatment options within the socioeconomic environment​ [94,95]. Addressing these gaps could integrate microbiome science into primary care and revolutionize cardiovascular treatment through enhanced professional health training, standardized protocols, and cost-effective testing methods. 

Limitations

Despite the growing interest in the role of the microbiome in cardiovascular health, several limitations exist in the current narrative review. First, the heterogeneity of study designs and methodologies can lead to inconsistencies in findings, making it challenging to draw definitive conclusions. Second, most existing research is primarily observational, which limits the ability to establish causality between microbiome variations and cardiovascular outcomes. Additionally, the complexity of the microbiome, influenced by numerous factors such as diet, environment, and genetics, complicates the identification of specific microbial targets for intervention. Lastly, most studies focus on narrow aspects of microbiome influence, often neglecting the interplay with other biological systems, which may restrict a comprehensive understanding of its role in cardiovascular health.

## Conclusions

This review explored the relationship between CVD and the gut microbiome, emphasizing the role of primary care interventions oriented to dietarian modifications. There is a relationship between gut dysbiosis and the progression of CVD that is evidenced by the production of TMAO, an organic component synthesized by the gut microbiome, that has been associated with endothelial dysfunction and endothelial stress, and it is utilized as a marker of cardiovascular dysfunction. Merging evidence suggests that targeted primary care interventions, including dietary modifications, probiotics, and lifestyle changes, can positively modulate the microbiome, thereby reducing cardiovascular risk. Integrating microbiome-focused strategies into routine primary care offers a promising avenue for personalized medicine, enhancing patient outcomes through tailored approaches. Further research is needed to refine these interventions and fully understand the complex interactions between the microbiome and cardiovascular health, ultimately paving the way for more effective prevention and treatment strategies. 
